# *Chlamydia trachomatis* TmeA promotes pedestal-like structure formation through N-WASP and TOCA-1 interactions

**DOI:** 10.1128/msphere.00101-25

**Published:** 2025-04-15

**Authors:** Alix McCullough, C. A. Jabeena, Steve Huang, Brianna Steiert, Robert Faris, Mary M. Weber

**Affiliations:** 1 Department of Microbiology and Immunology, University of Iowa Carver College of Medicine 12243 , Iowa City, Iowa, USA; 2 Department of Veterinary Microbiology and Pathology, Washington State University 6760, Pullman, Washington, USA; The University of Texas Medical Branch at Galveston, Galveston, Texas, USA

**Keywords:** *Chlamydia trachomatis*, T3SS, invasion, TmeA, TOCA-1, N-WASP, pedestal

## Abstract

*Chlamydia trachomatis* (*C.t*.) is the causative agent of several human diseases, including the sexually transmitted infection chlamydia and the eye infection trachoma. As an obligate intracellular bacterial pathogen, invasion is critical for establishing infection and subsequent pathogenesis. During invasion, *C.t*. secretes effector proteins via its type III secretion system (T3SS), which manipulate host actin cytoskeletal regulation to promote bacterial entry. Previous studies identified the T3SS effector protein TmeA as a key factor in *C.t*. invasion, as it recruits and activates N-WASP. This interaction, in turn, activates the Arp2/3 complex, driving cytoskeletal rearrangements at the invasion site to drive *C.t*. uptake. In this study, we define the role of the N-WASP CRIB domain in mediating this interaction and demonstrate that TmeA functions as a mimic of Cdc42 as part of its established role in activating N-WASP. Additionally, we identified TOCA-1 as another host protein that directly interacts with TmeA. In other bacterial pathogens, notably an enterohemorrhagic *E. coli*, N-WASP and TOCA-1 are hijacked to mediate pedestal formation. Using siRNA-mediated knockdown of N-WASP and TOCA-1, followed by transmission electron microscopy, we found that both proteins are important for *C.t*.-mediated pedestal-like structure formation. Collectively, these findings expand our understanding of the intricacies of *C.t*. invasion, highlighting how TmeA-mediated interactions with N-WASP and TOCA-1 contribute to pedestal-like structure formation, which may represent an early step in *C.t*. infection.

**IMPORTANCE:**

*Chlamydia trachomatis* (*C.t.*) is an obligate intracellular bacterial pathogen that poses a significant threat to human health, being associated with various diseases, including chlamydia—the most prevalent bacterial sexually transmitted infection—and trachoma. Although often asymptomatic, chlamydia infections can lead to severe complications, such as infertility, ectopic pregnancy, and an increased risk of cervical and ovarian cancers. As an intracellular pathogen, host cell invasion is critical for *C.t.* survival and pathogenesis. In this study, we provide new insights into the interactions between the *C.t.* invasion effector protein TmeA and the host proteins N-WASP and TOCA-1, revealing that both host proteins are involved in pedestal-like structure formation during early stages of *C.t.* infection. These findings deepen our understanding of the mechanisms underlying TmeA-mediated host cell invasion and highlight a key pathway contributing to *C.t.*-mediated pathogenesis.

## INTRODUCTION

*Chlamydia trachomatis* (*C.t*.) is an obligate intracellular bacterial pathogen that causes a wide range of diseases in humans, including trachoma and the sexually transmitted infection chlamydia (1, 2). While *C.t*. infections are often asymptomatic, complications from infection can lead to scarring of the female genital tract, resulting in pelvic inflammatory disease, infertility, ectopic pregnancy, and an increased risk of cervical and ovarian cancers (3, 4). Reinfections are common due to lack of long-term immunity and the absence of a vaccine (5). Understanding the molecular mechanisms by which *C.t*. invades epithelial cells and causes disease is vital for the development of improved therapeutics and identification of vaccine candidates.

As an obligate intracellular pathogen, invasion is a critical step in the lifecycle of *C.t*. As such, it has developed multiple mechanisms to invade host cells. At the end of its developmental cycle, *C.t*. prepackages a subset of type III secreted (T3S) effector proteins—including invasion-specific effector proteins translocated actin recruiting phosphoprotein (TarP), translocated early phospho-protein (TepP), translocated membrane effector A (TmeA), and translocated membrane effector B (TmeB)—into the elementary body (EB), the infectious form of the bacteria, to prime for new rounds of infection (6–10).

TarP directly binds F- and G-actin to facilitate actin filament bundling and elongation. It also interacts with host effectors to stimulate Rac1 signaling pathways and promotes actin branching through the Arp2/3 complex (11–15). Notably, TarP localizes to *C.t*.-associated pedestal-like structures, though its role in their formation has yet to be explored (6). TmeB, on the other hand, inhibits the Arp2/3 complex, suggesting a potential role in the disassembly of TmeA- and/or TarP-generated actin structures during invasion (16).

TepP plays a role in modulating the innate immune response during early infection by dampening the type I IFN response. Specifically, TepP recruits and activates the signaling adaptor CrkL and class I phosphoinositide 3-kinases (PI3K) at the nascent inclusion membranes, leading to generation of phosphoinositide-(3-5)-triphosphate (PIP3) (8, 17). Additionally, TepP reduces neutrophil recruitment in organoid models and disrupts epithelial cell–cell junctions through interactions with EPS8 (18, 19).

TmeA interacts with the host protein AHNAK; however, the functional significance of this interaction during infection remains unclear and appears to be distinct from TmeA's role in invasion (20). TmeA also recruits and activates the host protein Neural Wiskott-Aldrich syndrome protein (N-WASP), thereby activating the Arp2/3 complex, resulting in actin remodeling (21, 22). TmeA-mediated N-WASP recruitment stimulates *C.t*. uptake independent of TarP, though it also contributes to a TarP-mediated invasion pathway through oligomerization of host dynamin-2 (Dyn2) (23).

**Fig 1:**
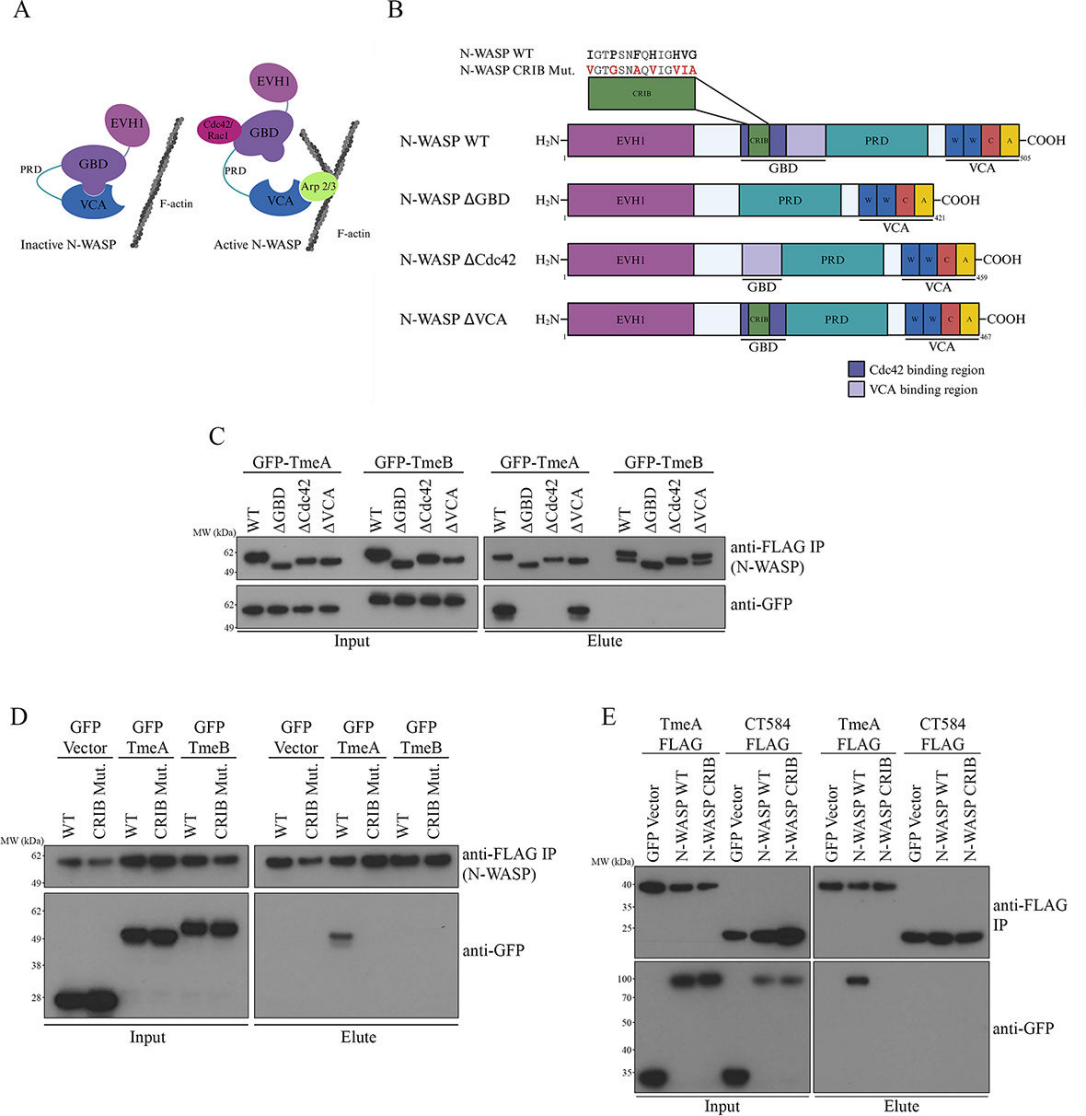
TmeA binds to the Cdc42 binding site of N-WASP. (A) Schematic depicting N-WASP in its autoinhibited and activated states. (B) Schematic depicting N-WASP deletion constructs. (C and D) FLAG-tagged N-WASP constructs were co-transfected with GFP-tagged *C.t*. effectors in HeLa cells. The FLAG-tagged proteins were immunoprecipitated, and samples were probed with anti-GFP or anti-FLAG antibodies. Data are representative of three biological replicates. (E) HeLa cells were transfected with GFP-tagged N-WASP WT or N-WASP CRIB mutant and infected with FLAG-tagged TmeA or CT584-expressing *C.t*. under aTc induction. The FLAG-tagged proteins were immunoprecipitated, and samples were probed with anti-GFP or anti-FLAG antibodies. Data are representative of two biological replicates.

N-WASP is modulated by multiple bacterial pathogens, including *Chlamydia pneumoniae*, *Salmonella enterica* Typhimurium, *Brucella abortus*, *Shigella flexneri*, and enterohemorrhagic *Escherichia coli* (EHEC) (24–29). N-WASP is an autoinhibited protein, with its GTPase binding domain (GBD) binding to and inhibiting the activity of its C-terminal verproline-cofilin-acidic region (VCA domain) (30). Upon binding of small GTPases, primarily Cdc42, to the Cdc42/Rac interacting binding region (CRIB) motif in the GBD, N-WASP is relieved of autoinhibition, thereby exposing the VCA domain. This exposure enables N-WASP to bind to the Arp2/3 complex, leading to actin branching and the formation of host cell structures such as filopodia and *E. coli* pedestals (Fig. 1A) (28–33). N-WASP also forms complexes with other host proteins including WASP-interacting protein (WIP) and transducer of Cdc42-dependent actin assembly (TOCA-1), which modulate its activity (34, 35). WIP regulates N-WASP activity and stabilizes actin filaments, while TOCA-1 works with Cdc42 to activate the N-WASP/WIP complex (34, 35).

EHEC employs a type III secretion system (T3SS) effector, EspF-like protein encoded on prophage U (EspF_u_), to recruit and activate N-WASP, facilitating pedestal formation (28, 29). Notably, it also recruits and activates TOCA-1, which is important for EHEC pedestal formation (31). Similarly, *S. flexneri* recruits TOCA-1 to its actin cocoons (36, 37). Given TmeA's role in N-WASP recruitment and modulation, as well as the prior identification of *C.t*.-associated pedestal-like structures, we hypothesized that TOCA-1 may also play a role in *C.t*. infection (6, 21, 22, 38).

Here, we further characterize the TmeA-N-WASP interaction, demonstrating that TmeA binds N-WASP via its CRIB motif. We also identify TOCA-1 as a host protein directly bound by TmeA, independent of TmeA-N-WASP interaction. Lastly, we establish a role for both N-WASP and TOCA-1 in *C.t*.-induced pedestal-like structure formation. Together, these findings provide new insights into the molecular interactions underlying *C.t*.-induced pedestal-like structure formation and highlight the multifaceted role of TmeA in manipulating host actin dynamics.

## RESULTS

### TmeA binds to the Cdc42 binding site of N-WASP

Our previous study demonstrated that the GTPase binding domain (GBD) ligand motif of TmeA is important for its interaction with N-WASP, and Keb et al. showed that TmeA can directly activate N-WASP (21, 22). However, TmeA's specific binding site on N-WASP has not been identified. Given the importance of the GBD ligand motif for this interaction—and that this ligand motif is used by EspF_u_ to bind to the autoinhibitory VCA binding site of the N-WASP GBD—we hypothesized that TmeA might similarly target the VCA binding site on N-WASP (Fig. 1A). To test this, we designed FLAG-tagged N-WASP deletion constructs targeting the entire GBD (ΔGBD, amino acids 191–275), the Cdc42 binding site within the GBD (ΔCdc42, amino acids 191–237), and the autoinhibitory VCA domain binding site within the GBD (ΔVCA, amino acids 215–275) (Fig. 1B). Cells were co-transfected with the N-WASP deletion constructs and GFP-tagged TmeA or GFP-TmeB as a negative control, and the FLAG-tagged protein was immunoprecipitated. Using this approach, we determined that the Cdc42 binding region, rather than the VCA-binding region, is necessary for TmeA-N-WASP interaction (Fig. 1C).

To further investigate whether TmeA binds the same site as Cdc42, we introduced conservative amino acid substitutions into the N-WASP CRIB motif (**I**GT**P**SN**F**Q**H**IG**HVG** → **V**GT**G**SN**A**Q**V**IG**VIA**). The CRIB motif is found in various Cdc42/Rac-activated effectors, including N-WASP, and is necessary but not sufficient for Cdc42 binding (39). Here, we demonstrate that the CRIB motif is required for TmeA-N-WASP interactions, as ectopically expressed TmeA failed to co-immunoprecipitate (co-IP) with the N-WASP CRIB mutant (Fig. 1D).

Because the previous experiments were performed with ectopically expressed plasmids rather than TmeA secreted from *C.t*., we sought to confirm the interaction occurs in the context of infection. Cells, transfected with GFP-N-WASP WT or CRIB constructs, were infected with *C.t*. expressing FLAG-tagged TmeA or CT584 as a negative *C.t*. effector control (40). Similar to ectopically expressed TmeA, TmeA secreted from *C.t*. specifically bound to N-WASP WT but not the CRIB mutant (Fig. 1E). These results confirm that TmeA mimics Cdc42 by binding to the N-WASP CRIB motif during infection, reinforcing its role as a functional mimic.

### TmeA interacts with endogenous TOCA-1

**Fig 2:**
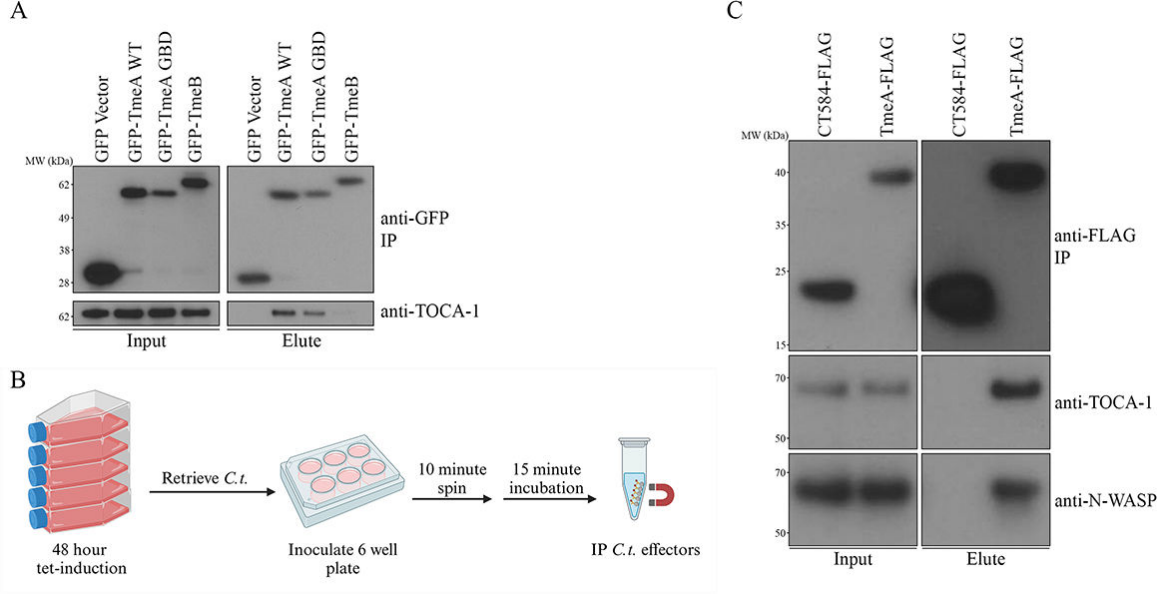
TmeA interacts with endogenous TOCA-1. (A) GFP-tagged *C.t*. effectors were transfected in HeLa cells. The GFP-tagged proteins were immunoprecipitated, and samples were probed with anti-GFP and anti-TOCA-1 antibodies. Data are representative of three biological replicates. (B) Schematic depicting a 15 minute infection-IP experiment. (C) HeLa cells were infected with *C.t*. expressing TmeA-FLAG or CT584 FLAG for 15 minutes, followed by immunoprecipitation. Samples were probed with anti-FLAG, anti-N-WASP, and anti-TOCA-1 antibodies. Data are representative of two biological replicates.

Given TOCA-1's role in N-WASP activation and its recruitment to EHEC pedestals, as well as its association with *Shigella flexneri* actin tails and cocoons alongside N-WASP (28, 29, 31, 36, 37), we hypothesized that TmeA may also interact with TOCA-1. To test this, we transfected HeLa cells with GFP-TmeA, a TmeA GBD ligand motif mutant (**LA**TH**I**QSK**L** → **VS**TH**V**QSK**V**) deficient in N-WASP binding (21), TmeB, or a GFP vector control and assessed binding to endogenous TOCA-1 using immunoprecipitation (Fig. 2A). Both TmeA and the TmeA GBD mutants co-immunoprecipitated with TOCA-1, indicating that TmeA interacts with TOCA-1 independently of its binding interactions with N-WASP.

To confirm that the TmeA-TOCA-1 interaction occurs during infection, we performed an invasion-stage specific IP. FLAG-tagged TmeA or CT584 was induced in *C.t*., after which EBs were isolated and used to infect fresh HeLa monolayers for 15 minutes (Fig. 2B). Following infection, we immunoprecipitated and assessed TmeA-FLAG and CT584-FLAG for binding to endogenous N-WASP and TOCA-1. Both N-WASP and TOCA-1 co-immunoprecipitated with TmeA (Fig. 2C), but not CT584, demonstrating that TmeA-TOCA-1 interactions occur at early stages of infection.

### TmeA directly binds TOCA-1, independent of N-WASP and Cdc42 binding sites

**Fig 3:**
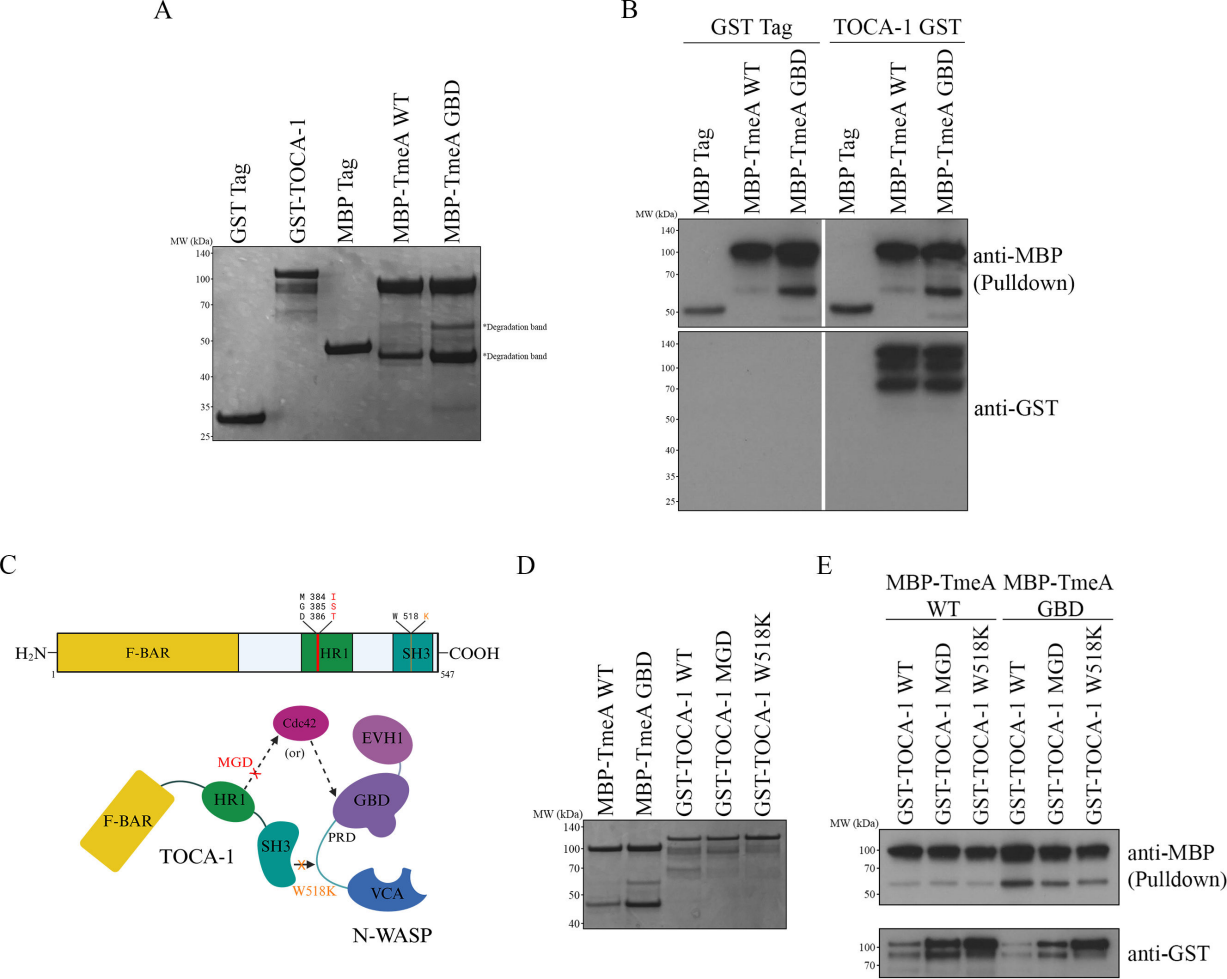
TmeA directly binds TOCA-1 independent of Cdc42 and N-WASP binding sites. (A and D) GST-tagged TOCA-1 constructs and MBP-tagged TmeA were expressed in *E. coli* and purified using GST or MBP resin on gravity columns. Recombinant protein expression was confirmed with Coomassie staining. (B and E) Recombinant MBP-tagged constructs were bound on MBP agarose. Bound proteins were incubated with GST-tagged TOCA-1 constructs and eluted, and samples were probed with anti-GST and anti-MBP antibodies. Data are representative of two replicates. (C) Schematic depicting known TOCA-1 mutants and their effect on binding to Cdc42 and N-WASP.

To confirm that TmeA directly binds to TOCA-1, we expressed and purified MBP-TmeA, MBP tag, GST tag, and GST-TOCA-1 (Fig. 3A) and performed a recombinant MBP pulldown assay using the GST-tagged proteins. We found that TmeA specifically and directly binds to TOCA-1 (Fig. 3B), confirming a direct interaction between TmeA and TOCA-1 that is independent of other host proteins or protein complexes.

To determine the specific binding site for TmeA on TOCA-1, we investigated whether known Cdc42 or SH3 binding sites on TOCA-1 play a role in direct TmeA-TOCA-1 interactions. TOCA-1 binds Cdc42 through its HR1 domain and interacts with N-WASP via its SH3 domain (35) (Fig. 3C). To test this, we performed a pulldown assay using MBP-tagged TmeA WT or TmeA GBD mutant, alongside TOCA-1 WT, a previously identified MGD mutant (lacking GTPase binding activity), and a previously identified W518K mutant (lacking SH3 binding activity) (35) (Fig. 3C through E). Both mutants were pulled down by TmeA WT and TmeA GBD mutant, demonstrating that neither the Cdc42 nor the N-WASP binding sites are required for TmeA-TOCA-1 interactions (Fig. 3E). Taken together, our findings indicate that TmeA directly interacts with TOCA-1, independently of its binding to N-WASP. Furthermore, we show that this interaction does not require TOCA-1's canonical Cdc42 or N-WASP binding motifs.

### N-WASP and TOCA-1 play a role in *C.t*.-mediated pedestal-like structure formation

**Fig 4:**
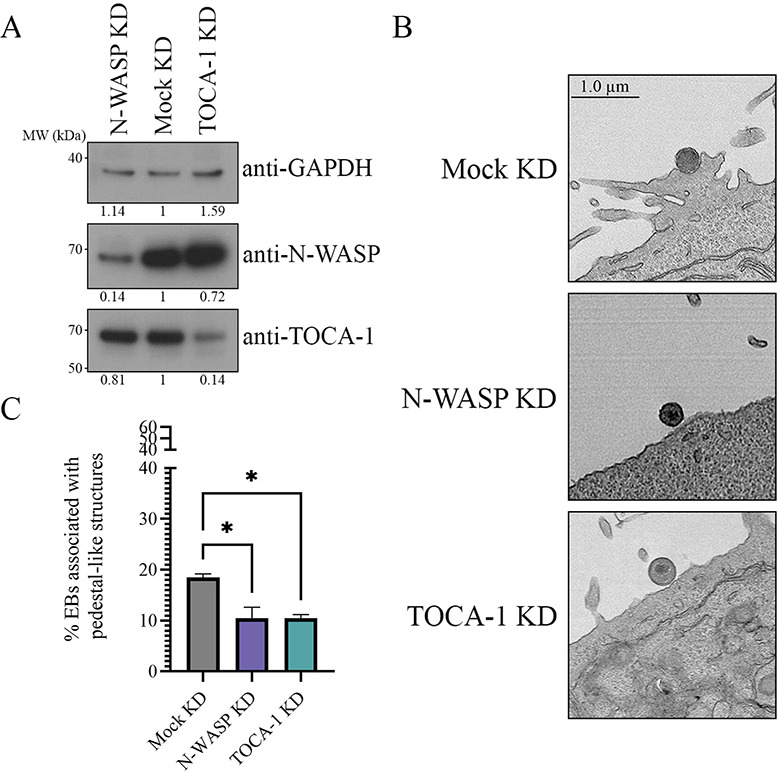
N-WASP and TOCA-1 play a role in *C.t*. pedestal-like structure formation. (A) N-WASP and TOCA-1 were knocked down in HeLa cells. Knockdown was verified using Western blotting, probing with anti-GAPDH, anti-N-WASP, and anti-TOCA-1 antibodies and quantified using densitometry, with relative density or adjusted relative density shown under the blots. Relative density was determined compared to mock KD, and relative density was adjusted for the N-WASP and TOCA-1 blots compared to the GAPDH standards. (B) HeLa cells were asynchronously infected with WTL2 at an MOI of 50 for 15 minutes and imaged with transmission electron microscopy; three representative images are shown. (C) Quantification of EBs associated with pedestal-like structures. A total of 100 EBs per experiment were assessed from two separate experiments, in which images were blinded and categorized as associated or not associated with pedestal-like structures. EBs associated with pedestal-like structures were compared to total EBs to determine the percentage associated with pedestal-like structures. Data represent the mean of two biological replicates. Error bars represent SD, *P < 0.05. Significance was determined using one-way ANOVA followed by Tukey’s multiple comparisons test.

*C.t*. has previously been shown to form pedestal-like structures during invasion of host cells (6, 38). Given TOCA-1's role in EspF_u_-mediated pedestal formation, we hypothesized that TOCA-1 may play a role in the formation of *C.t*. pedestal-like structures. To test this, we knocked down (KD) the expression of TOCA-1 and N-WASP using siRNA and subsequently challenged KD or mock KD control HeLa cells with WT L2 *C.t*. for 15 minutes (Fig. 4A). Transmission electron microscopy (TEM) was used to evaluate structures associated with EBs at the host cell surface (Fig. 4B; Fig. S1). Compared to the mock KD control, EBs on both N-WASP and TOCA-1 KD cells exhibited a significant reduction in association with pedestal-like structures (Fig. 4C), suggesting that both N-WASP and TOCA-1 play a role in *C.t.-*mediated pedestal-like formation.

## DISCUSSION

TmeA is an important T3SS effector protein that interacts with several cytoskeletal proteins to promote *C.t*. infection. Its interaction with the host protein AHNAK inhibits actin bundling, though this interaction does not appear to be essential for *C.t*.-mediated invasion, and its broader role during *C.t*. infection remains unclear (20). During the invasion phase, TmeA interacts with N-WASP, recruiting it to the invasion site to activate the Arp2/3 complex to promote actin branching (21, 22). Given compelling evidence that TmeA can directly activate N-WASP (22), we aimed to identify its precise binding site on N-WASP to gain deeper mechanistic insight into this interaction.

**Fig 5:**
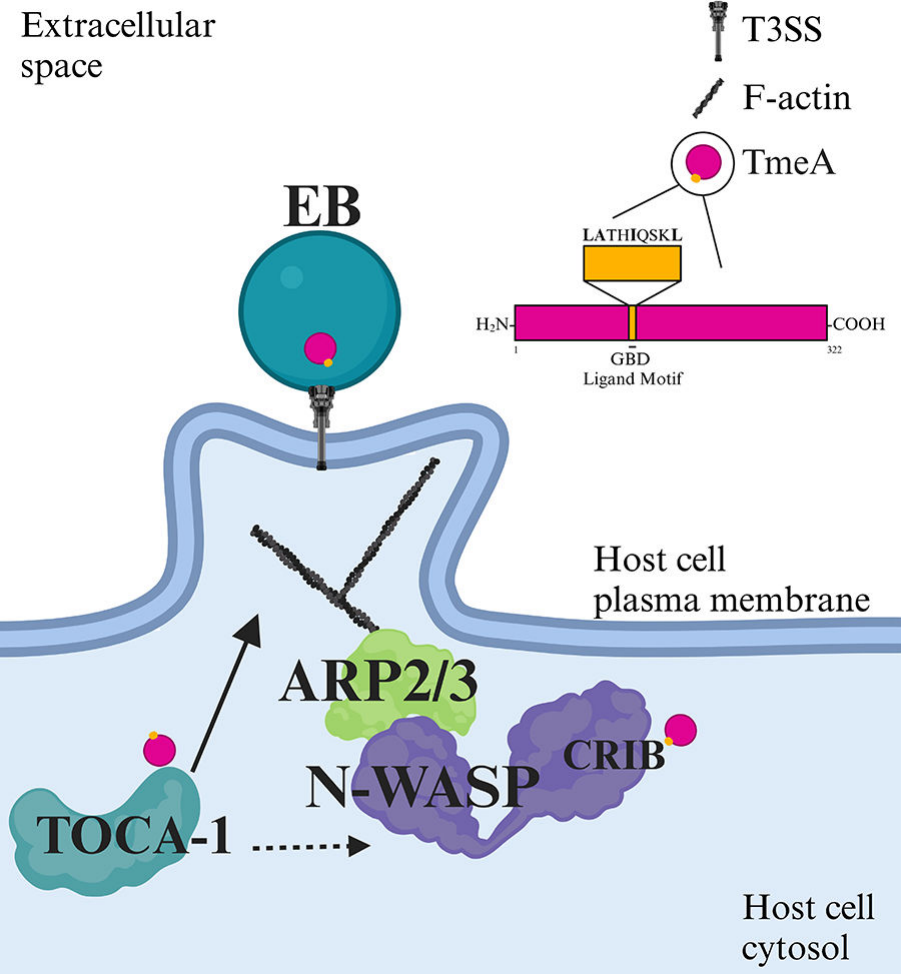
TmeA interacts with N-WASP and TOCA-1, which leads to EB pedestal-like structure association. TmeA interacts with N-WASP via the N-WASP CRIB domain as a Cdc42 mimic. It additionally interacts with TOCA-1 independently of N-WASP. The convergent roles of N-WASP and TOCA-1 in EB pedestal-like structure association indicate that these interactions likely contribute to a TmeA-mediated invasion pathway where EBs are taken up via pedestal-like structures.

Other bacterial effectors, such as *Shigella flexneri* IcsA and enterohemorrhagic *E. coli* EspF_u_, bind the VCA-binding region of the N-WASP GBD, while *Chlamydia pneumoniae* SemD acts as a molecular mimic of Cdc42 and binds the N-WASP CRIB domain (24, 29, 41). Prior work identified a GBD ligand motif in TmeA that is essential for co-immunoprecipitation with N-WASP (21), and additional studies demonstrated that TmeA is sufficient for N-WASP activation *in vitro (22*). These two key observations lead us to hypothesize that TmeA likely binds the VCA-binding region of the N-WASP GBD during *C.t*. invasion. However, our new data oppose this hypothesis, instead showing that, like SemD, TmeA requires the N-WASP CRIB motif for co-IP, while the VCA-binding region of the GBD is dispensable for this interaction. These results indicate that TmeA activates N-WASP by binding to the Cdc42 binding site, acting as a Cdc42 mimic to directly trigger N-WASP activation (Fig. 5). Our findings are supported by previous studies demonstrating that Rac, but not Cdc42, is activated and recruited to the site of *C.t*. invasion (42). Furthermore, TmeA is sufficient to activate N-WASP and the downstream Arp2/3 complex, leading to actin polymerization *in vitro*, even in the absence of Cdc42 (22). However, other studies have reported that Cdc42 is recruited to the invasion site and contributes to TmeA-mediated invasion, as its knockdown results in an invasion defect in *C.t*. expressing TmeA (22, 43). Further research is needed to determine the specific role of Cdc42 in TmeA-mediated invasion, though our new data build upon previous findings to establish TmeA as a direct activator of N-WASP via the CRIB motif. As these experiments were conducted using ectopically expressed N-WASP, future studies using recombinant N-WASP and CRIB mutants in actin polymerization assays will help us to further establish these direct interactions.

TOCA-1 is an interacting partner of N-WASP that is notably targeted by several bacterial effector proteins (31, 36, 37). TOCA-1-N-WASP interactions play a role in endocytic membrane trafficking (44, 45). The F-BAR domain of TOCA-1 binds to membranes and induces membrane curvature, as well as recruits N-WASP to the membrane (46). TOCA-1 also binds Cdc42 through the TOCA-1 HR1 domain and interacts via its SH3 domain with N-WASP (35). Analysis of binding dynamics revealed that Cdc42 has a significantly higher affinity for N-WASP than for TOCA-1, suggesting that TOCA-1 may facilitate N-WASP activation by “handing off” Cdc42 to N-WASP (47). Notably, these studies were conducted using only the purified TOCA-1 HR1 domain alone, and a tertiary complex between TOCA-1 HR1, N-WASP GBD, and Cdc42 was not observed. However, Förster resonance energy transfer (FRET) studies suggest potential tertiary complex formation, which may stabilize the flexible proline-rich domain (PRD) of N-WASP through interactions with the TOCA-1 SH3 domain (35, 45).

Both TOCA-1 and N-WASP have been linked to key cellular processes including endocytosis (44, 45), EHEC pedestal formation (31), and *Shigella flexneri* actin cocoon formation (36, 37). Given these roles, we explored whether TOCA-1 similarly contributes to *C.t*. infection. Our findings indicate that TmeA interacts with endogenous TOCA-1 during the early stages of infection. Recombinant TmeA and TOCA-1 directly bind, and these interactions are not abrogated by TOCA-1 mutants deficient in Cdc42 or N-WASP binding in either ectopically expressed or recombinant protein assays. We were unable to generate a TmeA truncation mutant deficient in TOCA-1 binding (Fig. S2), suggesting that TmeA might bind to multiple binding sites on TOCA-1. This hypothesis is supported by Alphafold 2.0 and HDOCK modeling, which predict interactions at multiple sites on both proteins (Fig. S2). Future studies will determine whether TmeA can bind to TOCA-1 and N-WASP simultaneously and whether it exhibits differing affinities for either protein, similar to Cdc42. Additionally, further investigation is needed to define TOCA-1's role during early stages of *C.t*. infection, as our current studies are limited to protein interactions and fixed-cell microscopy.

Our data suggest that N-WASP and TOCA-1 contribute to *C.t.-*associated pedestal-like structure formation, as knockdown of either protein significantly reduced EB-associated pedestal-like structures. Interestingly, TarP has also been implicated in *C.t.-*associated pedestal-like structures (6), raising the possibility that TmeA and TarP co-contribute to the formation of these structures. New work proposes a model of Dyn2-dependent *C.t*. invasion, where TarP recruits and TmeA activates Dyn2 (23). Dyn2 has been implicated in enteropathogenic *E. coli* pedestal formation alongside N-WASP (48). Thus, it is interesting to speculate that *C.t*. pedestal-like structures form through a collaborative pathway involving TmeA and TarP. Further studies should employ high-resolution live cell microscopy approaches to directly observe formation of these pedestal-like structures, *C.t*. internalization, and recruitment of host proteins.

In EHEC, actin pedestals are not used for host cell invasion but rather for attachment to the intestinal mucosa (49). This attachment is important for preventing bacterial clearance from the colon and may also facilitate actin-based motility and cell-to-cell spread (49–51). Additionally, pedestal formation may enhance the efficiency of secreted effector translocation into host cells, though this effect may be due to bacterial attachment rather than pedestal formation itself (52, 53). Notably, *C.t*. pedestal-like structure formation lacks the strong actin enrichment characteristic of EHEC pedestals (data not shown). This suggests that actin dynamics in *C.t*.-associated structures differ from those in EHEC. We propose that these differences stem from the distinct pathogenic strategies of these bacteria. While EHEC *E. coli* utilizes its T3SS to induce prominent actin-enriched pedestals for host cell attachment, *Chlamydia* may rely on more transient interactions with host structures that do not require extensive actin remodeling. These differences likely reflect the unique effector proteins employed by each pathogen and their distinct cellular niches, highlighting an exciting avenue for future research. Interestingly, invasive EPEC employs a small GTPase-mediated invasion strategy via WxxxE motif-containing T3SS effectors that activate small GTPases to induce uptake by non-phagocytic cells (54). Though TmeA does not contain a WxxxE motif and functions through direct N-WASP activation, invasive EPEC may provide a relevant comparison for studying potential overlaps between *C.t*. pedestal-like structures and non-invasive EHEC.

Taken together, we demonstrate that TmeA-N-WASP interactions depend on the N-WASP CRIB domain, establishing TmeA as a Cdc42 mimic. We further show that TmeA directly interacts with TOCA-1, independent of its association with N-WASP. These interactions are critical for *C.t.-*mediated pedestal-like structure formation, as knockdown of either host protein significantly reduces EB-associated pedestal-like structures (Fig. 5). Overall, this work adds to the growing body of knowledge surrounding *C.t*. invasion mechanisms.

## MATERIALS AND METHODS

### Bacterial and cell culture

Wild-type and *tmeA-lx* (55) *Chlamydia trachomatis* serovar L2 (LGV 434/Bu) was propagated in HeLa 229 cells (American Type Culture Collection). Purification of EBs was performed using a Gastrografin gradient as previously described (56). HeLa cells were cultured at 37°C under 5% CO_2_ in RPMI 1640 supplemented with l-glutamine, 10% fetal bovine serum (FBS) (26140079; Thermo Fisher Scientific), sodium bicarbonate (25080094; Thermo Fisher Scientific), sodium pyruvate (11360070; Thermo Fisher Scientific), and 50 µg/mL gentamicin (15750078; Thermo Fisher Scientific).

### Cloning

For ectopic expression, TmeA, TmeA GBD (21), and TmeB were cloned into pcDNA3.1+N eGFP (Genscript) using KpnI/XbaI or NotI/XbaI sites. TmeA GBD truncations were generated from the TmeA GBD construct and cloned into pcDNA3.1+N eGFP (Genscript) using KpnI/NotI sites. pcDNA 3.1 FLAG N-WASP∆GBD (21), N-WASP∆Cdc42, N-WASP∆VCA, and N-WASP CRIB mutant were generated by GenScript. pCS2 +MT-hTOCA-1 WT, MGD, and W518K (Addgene 33030, 33033, and 33031) (35) were cloned into pcDNA 3.1 HA (GenScript) using KpnI/XhoI sites. For recombinant protein expression, TmeA and TmeA GBD were cloned into pMAL-c5VT (University of Iowa Protein and Crystallography Facility) using NotI/SalI sites. Codon-optimized pGEX 6P1 TOCA-1 WT, MGD, and W528K were purchased from GenScript.

### Transfection

HeLa cells were seeded at 4 × 10^5^ in six-well plates (10062-892; VWR) 24 hours prior to transfection. GFP-tagged TmeA, TmeA GBD, TmeB, or empty vector were transfected alone or co-transfected with FLAG-tagged N-WASP WT, N-WASP∆GBD (21), N-WASP∆Cdc42, N-WASP∆VCA, or N-WASP CRIB mutant. GFP-tagged TmeA GBD truncations were co-transfected with HA-tagged TOCA-1 WT. GFP-tagged N-WASP WT or N-WASP CRIB mutant was used for transfection-infection IPs. HeLa cells were transfected using Lipofectamine LTX (15338100; Thermo Fisher Scientific) per the manufacturer's instructions. When required, cells were infected at a multiplicity of infection (MOI) of 5 with serovar L2 *C.t*. expressing pBomb4-tet-TmeA-FLAG or pBomb4-tet-CT584-FLAG under aTc (anhydrous tetracycline) induction (10 ng/mL). Samples were incubated for 18 hours, followed by co-immunoprecipitation.

### Invasion-stage infection

HeLa cells were seeded into five T175 flasks (660175; Greiner) at 4.9 × 10^6^ cells per flask and infected at an MOI of 1 with serovar L2 *C.t*. expressing pBomb4-tet-TmeA-FLAG or pBomb4-tet-CT584-FLAG, and expression of the FLAG-tagged fusion was induced using 10 ng/mL aTc. At 48 h post-infection, flasks were washed with HyClone Cell Culture Grade water (SH3052902; Cytivia), lysed via scraping, and lysates were centrifuged at 1,500 RPM for 3 min to remove cellular debris. Supernatants were transferred to microcentrifuge tubes, and *C.t*. was pelleted via centrifugation at 12,000 × g for 20 min. *C.t*. was resuspended in RPMI with 10 ng/mL aTc and used to infect fresh HeLa cell monolayers in a six-well plate (10062-892; VWR). To synchronize the infection, we spun plates at 900 × g for 10 min at 15°C, followed by a 15 min incubation at 37°C. Cells were then lysed, and FLAG-tagged effectors were immunoprecipitated.

### Co-immunoprecipitation

Transfected and/or infected cells were washed with 1× PBS (10010023; Gibco) and lysed using Eukaryotic Lysis Solution (ELS) (50 mM Tris HCl, pH 7.5) (15567-027; Invitrogen), 150 mM NaCl (S23020; RPI), 1 mM EDTA (E57020; RPI), and 1% Triton X-100 (BP151-500; Thermo Fisher Scientific) with Halt protease inhibitor cocktail (78430; Thermo Fisher Scientific). Lysates were incubated on ice for 20 minutes, followed by pelleting of cell debris via centrifugation at 12,000 × *g* for 20 min at 4°C. Cell-free supernatants were applied to anti-GFP mAb-magnetic beads (D153-11; MLB) or anti-FLAG M2 magnetic beads (SLCQ2245; Sigma) for 2 h at 4°C, or anti-FLAG M2 Affinity Gel (A2220; Sigma) overnight at 4°C. Unbound proteins were removed by washing in ELS without Triton-X 100, and the GFP- or FLAG-tagged proteins were eluted using NuPAGE LDS Sample Buffer (NP0007; Thermo Fisher Scientific) and heated at 100°C for 5 min. Samples were analyzed by Western blotting.

### Western blotting

Supernatants and eluted samples were run on 4–12% SurePAGE Bis-Tris Gels (M00652, M00653, or M00654; GenScript), followed by wet transfer to Immobilon-P polyvinylidene difluoride (PVDF) membranes (IPVH0010; Sigma-Aldrich). Membranes were probed with primary antibodies against TOCA-1 (1:4,000, PA5-85726; Invitrogen), GFP (1:10,000, NB600-308; Novus Biologicals), FLAG (1:4,000, 701629; Invitrogen), or HA (1:4,000, H6908; Sigma-Aldrich) followed by HRP-conjugated secondary antibodies (1:10,000; rabbit: 1706515, BioRad; mouse: 31430, Thermo Fisher Scientific), then detected using ECL Prime Western Blotting Detection Reagent (RPN2236; Sigma-Aldrich) and X-ray film.

### Recombinant protein purification

Gglutathione S-transferase (GST) vector (pGEX6p1), Mmaltose-binding protein (MBP) vector (pMALc5V2), MBP-TmeA, and MBP-TmeA GBD were expressed in BL21 (DE3) *E. coli* and codon-optimized GST-TOCA-1 WT, GST-TOCA-1 MGD, and GST-TOCA-1 W518K were expressed in Rosetta (DE3) *E. coli* (70954; Novagen). Transformants were inoculated in 50 mL of Luria broth (LB) (L24400; RPI) and then incubated at 37°C overnight. For expression of MBP-tagged proteins, glucose (G32045; RPI) was added to LB broth at a concentration of 2 g/L. Overnight cultures were added to 950 mL LB broth and grown to OD 0.6–0.8. Protein expression was induced with 1 mM isopropyl-β-d-thiogalactopyranoside (IPTG) (I56000-25.0; RPI), followed by overnight induction at 18°C. *E. coli* was lysed in MBP (20 mM Tris HCl, pH 7.5, 200 mM NaCl, 1 mM EDTA, and 1 mM sodium azide [RTC000068; Sigma-Aldrich]; 10% glycerol [BP229-1; Thermo Fisher Scientific]; and 1 mM dithiothreitol [DTT] [D11000; RPI]) or GST (50 mM Tris HCl. pH 7.5, 500 mM NaCl, 10% glycerol, and 1 mM DTT) column/lysis buffer via sonication with one pulse on for 1 second and one pulse off for 1 second for 3 min at 70% amplitude. Lysed cells were pelleted at 10,000 × *g* for 30 min at 4°C and supernatants were collected. One milliliter of Pierce glutathione agarose (16100; Thermo Fisher Scientific) or amylose resin (E8021S; NEB) for GST- or MBP-tagged proteins, respectively, was added to 25 mL gravity columns and washed with the appropriate column/lysis buffer. Supernatants were incubated in columns at 4°C for 1 h for protein conjugation. The columns were then washed with 75 mL lysis/column buffer before addition of 2 mL elution buffer for GST-tagged (50 mM Tris HCl, pH 7.5, 150 mM NaCl, and 10 mM l-glutathione reduced [G22010; RPI]) or MBP-tagged [10 mM d-(+)-maltose monohydrate (M22000; RPI)] proteins. Columns were incubated with rotation for 30 min at 4°C and elutions were collected in 1 mL fractions, with a total of 4 fractions after two elutions. Protein concentration was determined via *A*_280_ measurement on a Nanodrop 2000 (Thermo Fisher Scientific), and protein purity was confirmed via Coomassie staining (1610436; Bio-Rad) of proteins on SDS-PAGE gels.

### Recombinant protein pulldown

Buffer exchange was performed on GST-tagged proteins (GST tag, GST-TOCA-1 WT, GST-TOCA-1 MGD, and GST-TOCA-1 W518K) and MBP-tagged proteins (MBP tag, MBP-TmeA, and MBP-TmeA GBD) using Amicon Ultra Centrifugal Filter Units (UFC901024; Millipore) to remove reduced glutathione or maltose, respectively. Proteins were added to the filters with 10 mL GST column/lysis buffer or MBP column buffer, followed by 4°C centrifugation at 4,000 × *g* for 15 min, for a total of five centrifugations. Protein concentration after buffer exchange was determined via *A*_280_ measurement on a Nanodrop 2000 (Thermo Fisher Scientific). We conjugated 40 µg of the MBP-tagged bait proteins to 0.5 mL amylose resin (E8021S; NEB), as described above (“Recombinant protein purification”), followed by five washes with appropriate column/lysis buffer. Forty micrograms of GST-tagged prey proteins was added to the columns and incubated overnight at 4°C with rotation. Columns were washed five times with MBP column/lysis buffer, and proteins were eluted as described above (“Recombinant protein purification”) (57). Eluted proteins were analyzed by Western blotting. Membranes were probed with primary antibodies against MBP (1:4,000, R29.6; Santa Cruz Biotech) or GST (1:4,000, 8-326; Invitrogen).

### Transmission electron microscopy

HeLa cells were seeded at 2.5 × 10^5^ in six-well plates 24 hours prior to siRNA knockdown. *FNBP1L* (TOCA-1) or *WASL* (N-WASP) expression was knocked down using DharmaFECT 1 (T-2001-03; Horizon Discovery) with SmartPool siRNA (*FNBP1L* 020718-01-0020, *WASL* L-006444-00-0020; Horizon Discovery) according to the manufacturer's protocol (Horizon Discovery). Knockdown was verified 48 h post-transfection by western blotting using primary antibodies, all at a concentration of 1:4000, against TOCA-1 (PA5-85726; Invitrogen), N-WASP (NBP1-82512; Novus Biologicals), or a GAPDH loading control (SAB4300645; Sigma-Aldrich). Knockdown was quantified using densitometry. Simultaneous to knockdown verification, remaining cells were asynchronously infected with WT L2 *C.t*. at an MOI of 50 for 15 min. Cells were fixed using 2.5% glutaraldehyde at 4°C overnight (21). Cells were post-fixed using 1% osmium tetroxide and 1.5% potassium ferrocyanide, followed by staining using 2.5% uranyl acetate. Samples were dehydrated using increasing concentrations of EtOH (50–100%) before embedding in Eponate12 resin (Ted Pella, Inc.). Using a Leica UC6 ultramicrotome (Leica Microsystems), 80 nm thin sections were made (21). Images were taken on a Hitachi HT7800 transmission electron microscope with an AMT NanoSprint15 high-resolution, high-sensitivity camera system. Two biological replicates of 100 EBs per sample were imaged, blinded, and scored for association with pedestal-like structures, filopodia-like structures, pit-like structures, or no cell structure utilizing example images of the structures from Faris et al. (21), Carabeo et al. (38), and Clifton et al. (6).

### Molecular docking of TmeA and TOCA-1

The tertiary structure of TmeA and TOCA-1 was predicted using AlphaFold 2.0 (58). The generated structures were used for protein docking using the Hdock server (59). The resultant docking file gave possible interface residue pairs between TmeA and TOCA-1 and a confidence score. Binding is very likely when the confidence score is above 0.7.

### Statistical analysis

One-way analyses of variance (ANOVAs) were used with Tukey's multiple comparisons post-test, with *P* < 0.05 (*). Analysis was performed using GraphPad Prism 9.4.1 software.

## SUPPLEMENTAL MATERIAL

Supplemental figures msphere.00101-25-s0001.docx Figures S1 and S2.

ASM does not own the copyrights to Supplemental Material that may be linked to, or accessed through, an article. The authors have granted ASM a non-exclusive, world-wide license to publish the Supplemental Material files. Please contact the corresponding author directly for reuse.
