# Supplementary material for: Chlamydia trachomatis TmeA promotes pedestal-like structure formation through N-WASP and TOCA-1 interactions
Source: mSphere. 2025 Apr 15;10(5):e00101-25. doi: 10.1128/msphere.00101-25 (PMC12108077; doi:10.1128/msphere.00101-25)
Supplement: Supplemental figures — Figures S1 and S2. [file msphere.00101-25-s0001.docx]

**
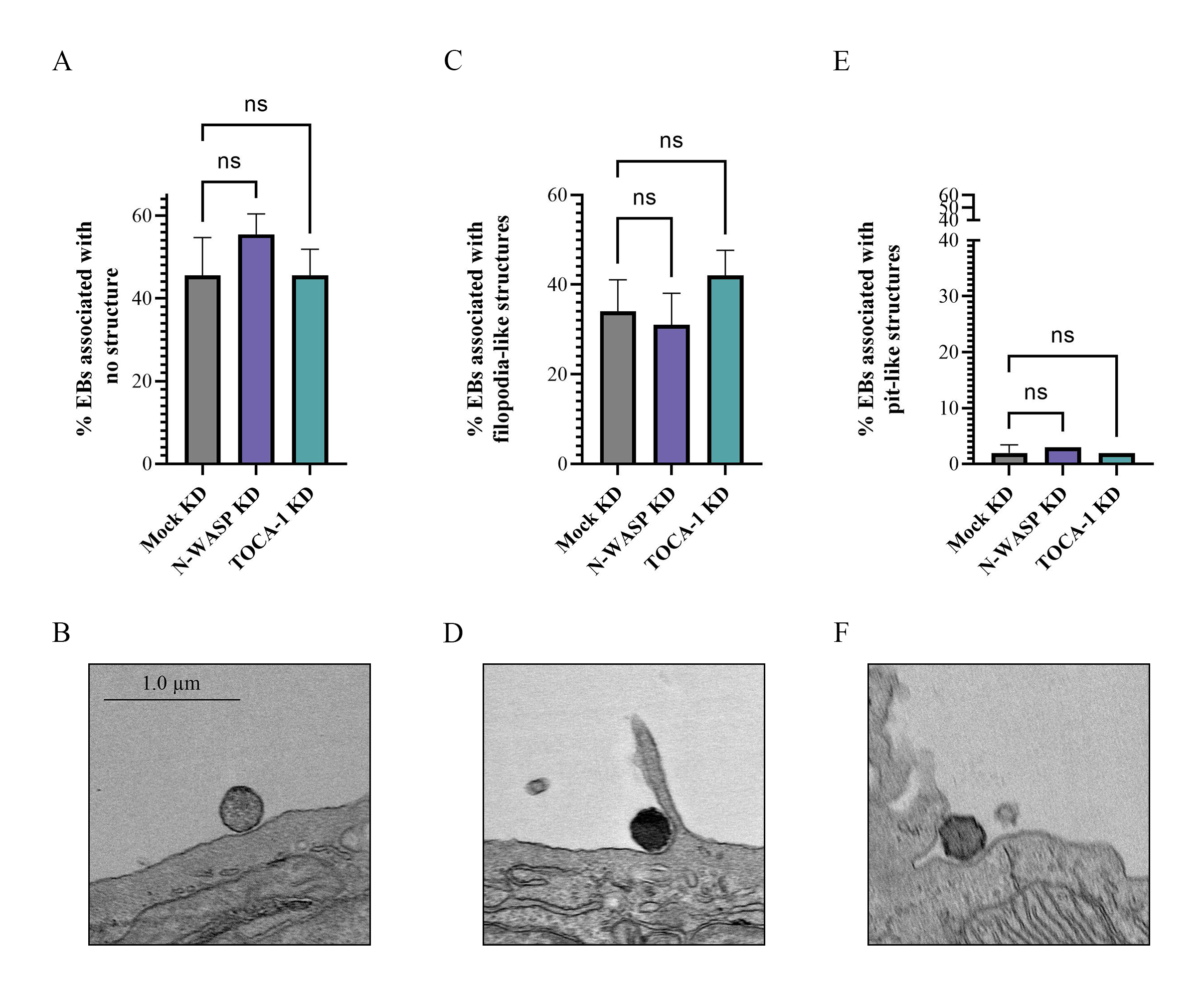
**

**Supplemental Figure 1. N-WASP and TOCA-1 KD do not impact formation of other structures.** Quantification and examples of EBs associated with **A,B.** no structure, **C,D.** filopodia or **E,F.** pits. 100 EBs per experiment were assessed from two separate experiments, in which images were blinded and categorized based on structure association. EBs in each category were compared to total EBs to determine the percentage associated with structures. EBs associated with each category were compared to total EBs to determine the percentage associated with the given category. Bars represent the mean of 2 biological replicates. Error bars represent SD, *P < 0.05. Significance was determined using one-way ANOVA followed Tukey’s multiple comparisons test. Data are representative of two biological replicates.


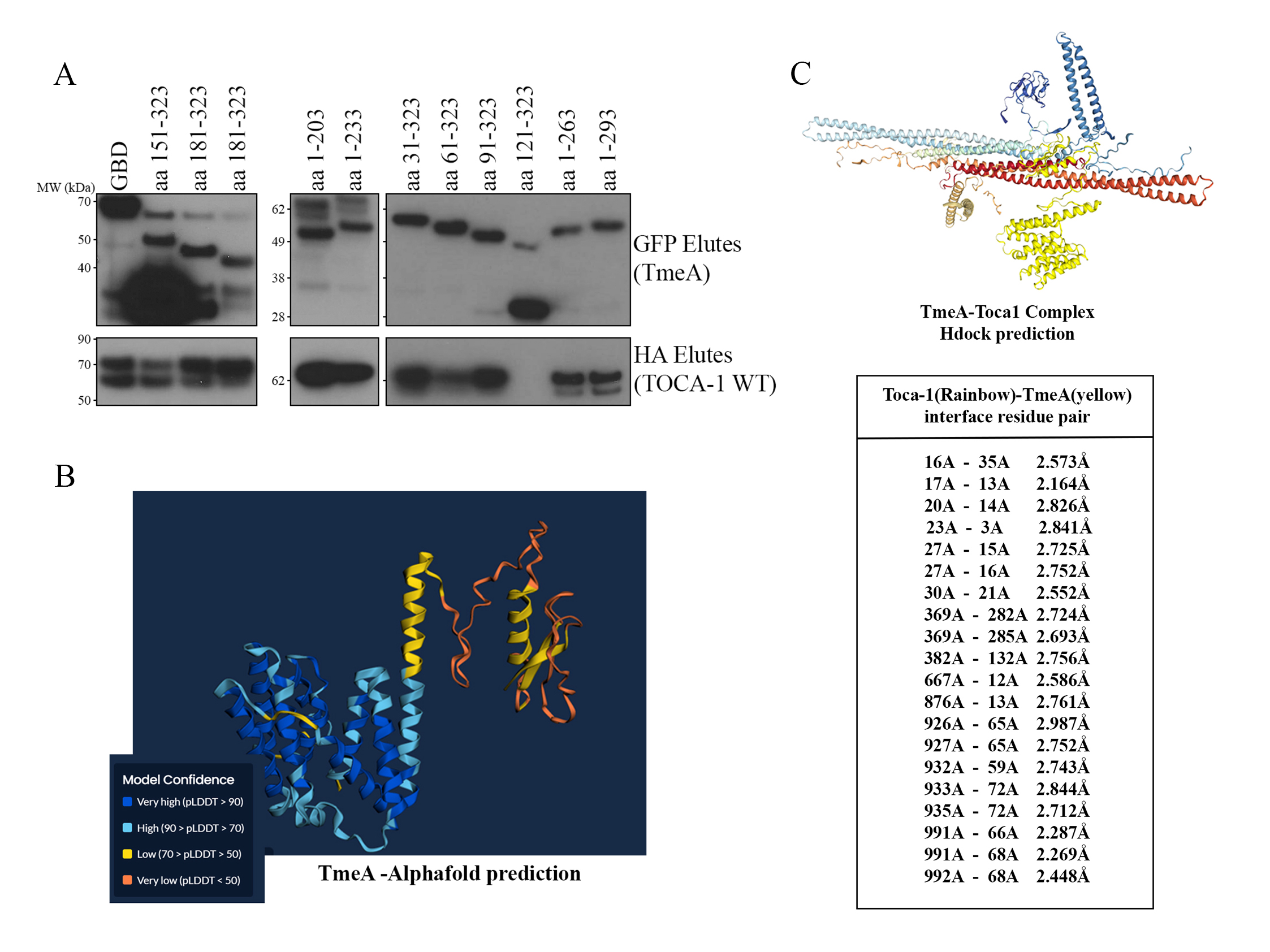


**Supplemental Figure 2. TmeA likely binds TOCA-1 at multiple binding sites. A.** GFP-tagged TmeA truncations were co-transfected with HA-tagged TOCA-1 WT in HeLa cells. The GFP tagged proteins were immunoprecipitated and samples were probed with anti-GFP or anti-HA antibodies. **B.** Alphafold modeling predicting tertiary structure of TmeA. **C.** Binding complex prediction of TmeA (yellow, ligand) and TOCA-1 (rainbow, receptor). Confidence score is 0.9777, thus TmeA and TOCA-1 are very likely to bind. Residue pairs with predicted distance < 3Å are noted.
